# Experimental study on the mechanical properties and creep development of loess grouting reinforcement material

**DOI:** 10.1098/rsos.250330

**Published:** 2025-08-06

**Authors:** Yuxi Guo, Yan Qin, Nengxiong Xu, Jiayu Qin, Wenjing Zhou, Hai Wang

**Affiliations:** ^1^ China University of Geosciences Beijing, Beijing, People's Republic of China; ^2^ Engineering and Technology Innovation Center for Risk Prevention and Control of Major Project Geosafety, Beijing, People's Republic of China; ^3^ Shanxi Transportation Planning Survey and Design Institute, Taiyuan, People's Republic of China

**Keywords:** Loess slurry material, Grouting reinforcement body, Mechanical properties, Goaf, Creep characteristics

## Abstract

The focus was on studying the effects of loess-based grouting material proportioning, fractured rock mass grading, overlying load, and grouting pressure on the grouting reinforcement effect of the fractured rock mass. The results showed that when the water-to-solid ratio was controlled at 1:1.4–1:1.0, the solid phase ratio was 3:7–5:5, and the water glass content was 3–5%, the slurry properties met the requirements for grouting treatment in the goaf. Through grouting reinforcement tests, it was found that as the strength of the grouting material increased, the crushing gradation decreased, the overlying load increased, the grouting pressure increased, and the difference in mechanical properties between the grouting material and the rock block decreased. The compression failure mode shows a ‘rock-like’ characteristic, with cracks mainly extending along the ‘rock-slurry-rock’ bonding surface, followed by extending along the grouting solid body. The instantaneous strain and axial creep values first decreased and then increased during the graded loading process, and the creep rate first decreased rapidly and then stabilized. Finally, the long-term strength of the grouting reinforcement material was determined to be 2.1 MPa, accounting for 65–80% of the instantaneous strength.

## 1. Introduction

With the continuous development of underground coal resources, the formation of a large number of goafs will lead to a series of geological disasters, such as surface subsidence and changes in groundwater levels [1,2]; filling goafs is an inevitable trend for the green development of mines [3]. At present, an unstable goaf is mainly controlled through grouting reinforcement [4,5]. Through filling, bonding, solidification and other effects of grouting materials, the fragmented rock mass is reinforced into a whole [6–8], improving the stress state and structural strength of the fractured rock mass in the normal direction of the fracture surface [7,9], thereby improving the overall stability of the goaf.

The selection and proportioning design of grouting materials are related to the physical and mechanical properties of grouting materials [10–12] and affect the quality of grouting engineering. At present, the slurry materials used for grouting treatment in goafs mainly include pure cement slurry, cement-fly ash slurry and cement-clay slurry [13,14]. Pure cement slurry injection is effective, and the early compressive strength of the grouting solid body can reach over 10 MPa, significantly exceeding the performance requirements of mine injection materials. However, the economic cost is high, and it is not suitable for large-scale use [15–17]. At present, the grouting treatment of goaf mainly adopts cement-fly ash slurry materials, and the addition of fly ash reduces the fluidity and early strength of the grouting material [18]. When the fly ash content is 0–30%, the requirements of grouting strength are met. When the fly ash content reached 30%, the permeability and resistance to chloride ion penetration of the grouting material were optimal. When the fly ash content reached 40%, the flowability, durability and compressive strength of the grouting material were optimal [18–20]. However, with increasing attention and intensity of environmental protection, heavy metals and toxic substances in fly ash grouting materials pose a threat to groundwater and soil. Therefore, there is an urgent need to develop low-cost, green and environmentally friendly grouting filling materials that meet the requirements of goaf treatment [17]. Loess is widely distributed worldwide, and loess-based grouting materials have advantages such as a wide source, low-cost, easy operation and similar composition to fly ash. Loess is mainly composed of SiO_2_ and Al_2_O_3_, which can react with CaO to produce cementitious products, such as hydrated calcium silicate and hydrated calcium aluminate. It can also react with the Ca(OH)_2_ generated by cement to reduce the alkali content of grouting materials and enhance their impermeability [21]. Therefore, loess can replace cement as a grouting material in the treatment of goafs. Gu *et al*. [22,23] studied the effects of factors such as water-to-solid ratio, solid-to-solid ratio and admixture dosage on the properties of loess-based grout materials. They found that as the loess content increased, the strength and fluidity of the grouting solid body decreased, the initial and final setting times were prolonged, and the coarser the loess particle size, the stronger was the ability of the grouting solid body to resist deformation. Wu *et al*. [24] studied the physical and mechanical properties of a cement-loess slurry using the full filling pressure grouting method. It is found that when the ratio of cement to loess is within the range of 3:7 to 4:6, the compressive strength of the grouted stone body is between 2.82 and 7.88 MPa, which meets the compressive strength requirements for grouted stone bodies in mined-out areas. Compared with other grouting materials, this method reduces material costs by 26–32%. The above research mainly focuses on the material properties of loess-based grout and its mechanical properties under pure grouting consolidation conditions. However, in the actual grouting project in the goaf, the loess-based grouting material will be combined with the broken rock mass in the goaf after injection to form a composite structure of loess-based grouting reinforcement material, and its mechanical behaviour will be different from that of pure loess-based grouting solid body. At present, the research on the strength and deformation characteristics of loess grouting reinforcement materials is not perfect, and it is urgent to carry out relevant research.

The strength of grouting reinforcement is a key indicator for evaluating the treatment effect after using the preferred grouting materials for treatment. At present, the evaluation of grouting reinforcement strength is mainly based on testing the consolidation strength of the grout. However, in the fractured rock mass that has been reinforced by grouting in the goaf, there are numerous rock-grout-rock structural interfaces [25–27] and the reinforcement effect cannot be evaluated solely based on the consolidation strength of the slurry. Fang *et al.* [28] established a mechanical model for the grouting reinforcement of fractured rock masses and analysed the influence of factors such as rock fragmentation degree, grouting material bonding performance and grouting pressure on solidification strength. They found that the main controlling factor affecting the strength of grouting reinforcement material is the grouting material bonding performance, followed by grouting pressure [29], and a calculation formula for predicting the compressive strength of the grouting reinforcement material was proposed [28,30]. Zong *et al*. [31] conducted uniaxial compression tests on fractured sandstone after grouting and determined the effects of grouting strength, deformation characteristics and failure mode on the grouting reinforcement material. They also found that the failure mode of the specimens was primarily plastic failure. Regarding the failure mode of grouting and solidification, Wang *et al*. [32–34] found through experiments that not only did the residual strength of the grouting reinforcement material increase significantly after grouting but the synchronization of the lateral and radial deformation of the grouting reinforcement material also increased, revealing the deformation mechanism of the transformation from grouting and solidification failure mode to plastic failure. Previous research on grouting reinforcement has mainly focused on the solidification properties of the grout material itself, including grout performance, optimal mix ratio and prediction of the solid strength. However, when grouting reinforcement is applied to fractured rock masses, the direct target of the grouting material is the void/fracture surface of the fractured rock mass [27,35], not only the solidification properties of the grouting material itself but also the reinforcement material after grouting often undergoes creep process under the overlying load. Therefore, as a combination of grouting material and fractured rock mass, the bonding performance of the void/fracture surface affects the mutual mechanical properties and creep characteristics of the grouting material and fractured rock mass. Moreover, under various grouting factors (grouting material ratio, fracture grading, overlying load and grouting pressure), the mechanical properties and creep characteristics are more complex. To study the grouting reinforcement mechanism of fractured rock masses, it is necessary to conduct systematic research on the mechanical properties, failure modes and creep characteristics of grouting reinforcement materials under various grouting factors.

The purpose of this study is to explore the feasibility of using loess-cement as a grouting material for mining and analysing the reinforcement mechanism of fractured rock mass grouting under different grouting factors, the failure mode of the ‘rock-slurry-rock’ structural plane and the creep characteristics. This study considered loess grouting reinforcement materials as the research object. The optimal grouting ratio that meets the requirements of goaf treatment is selected by conducting loess grouting material performance testing, fractured rock mass grouting reinforcement testing and solidification creep testing. The influence mechanism of the grouting material ratio, crushing gradation, overlying load and grouting pressure on the mechanical and creep characteristics of the grouting reinforcement material is revealed, and the failure mode of the grouting reinforcement material is elucidated. The research results provide a theoretical basis and reference for the application of grouting materials in loess mining and the improvement of grouting reinforcement mechanisms for fractured rock masses.

## 2. Material and methods

### 2.1. Experimental set-up

**Figure 1.**
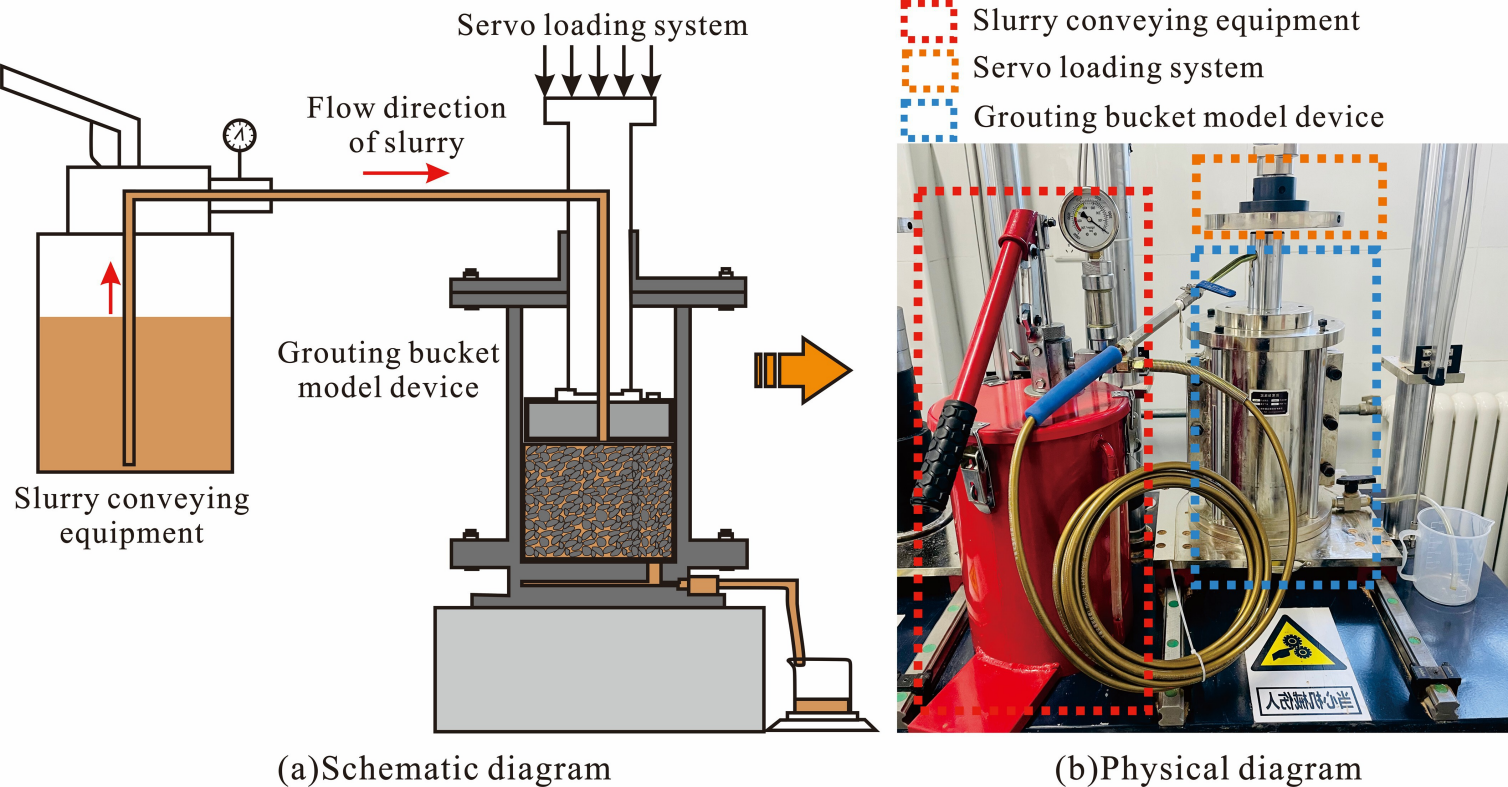
Grouting simulation test device.

The grouting simulation test device used in this study is shown in figure 1a, consisting of a grouting bucket model device, slurry conveying equipment and a servo loading system (figure 1b). This experimental device can simulate the injection of grout into discrete and fragmented rock masses using a grouting pump under the action of overlying loads. Through the filling, cementation, solidification and other effects of the grout, it is reinforced into a whole, forming a grouting reinforcement material composed of ‘rock-slurry-rock’.

#### 2.1.1. Grouting bucket model device

The grouting bucket was made of 45 # steel plate as a whole, and the device mainly included cylinder and pressure-head components (figure 1b). A containment chamber inside the cylinder assembly can be used to accommodate fractured rock masses. A part of the pressure-head component extends into the receiving chamber and can move up and down in the cylinder assembly by applying pressure to the fractured rock mass through a connection with the pressurization device. The pressure-head component was designed with a grouting channel, which was connected to the receiving chamber. The entrance of the grouting channel was used to connect grouting pumps.

#### 2.1.2. Slurry conveying equipment

The equipment used for conveying the slurry in the experiment consisted of a manual grouting pump, grouting pressure gauge and slurry conveying pipe manufactured by the Shanghai Jiadeli Company, as shown in figure 1b. Using a grouting pump to provide conveying power for the slurry, when the pressure reading reaches the specified pressure, the loess slurry material enters the grouting bucket through the grouting pipe to reinforce the fractured rock mass inside the device.

#### 2.1.3. Servo loading system

The model selected for this experiment was the KYSR-S servo loader. The servo loading system consists of four parts: a servo motor loading device, a servo motor loading control unit, a creep test bench and a displacement monitoring device, as shown in figure 1b.

### 2.2. Materials

#### 2.2.1. Loess soil

**Figure 2.**
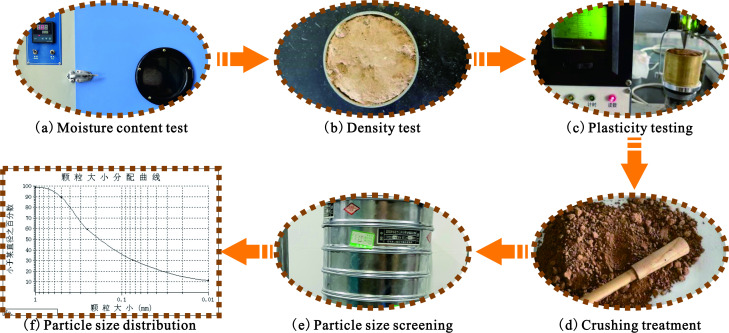
Basic physical property index testing of loess samples.

The loess used in this experiment was obtained from North China and subjected to drying, crushing and screening. The basic physical properties of the loess material were tested (figure 2), and the test results are listed in table 1.

**Table 1.** Basic physical property indicators of loess samples.

moisture content(%)	density (g/cm^3^)	liquid limit	plastic limit	plasticity index	liquidity index	soil quality
4.0	1.64	23.2	16.3	6.9	−1.78	silt

#### 2.2.2. Cement

The cement selected for the experiment was 425# ordinary Portland cement produced by Hongtai Factory in Luannan County, China, with a particle size range of 10−45 μm. The specific chemical compositions are listed in table 2.

**Table 2.** The main chemical components of ordinary Portland cement.

chemical composition	SiO_2_	Al_2_O_3_	CaO	Fe_2_O_3_	MgO	SO_3_	K_2_O
content /%	21.15	5.46	64.08	3.35	1.35	2.13	1.11

#### 2.2.3. Water glass

The experiment selected Na₂O·nSiO₂, produced by Shandong Duofeng Chemical in China, with a particle modulus of 2.4.

#### 2.2.4. Broken sandstone

**Figure 3.**
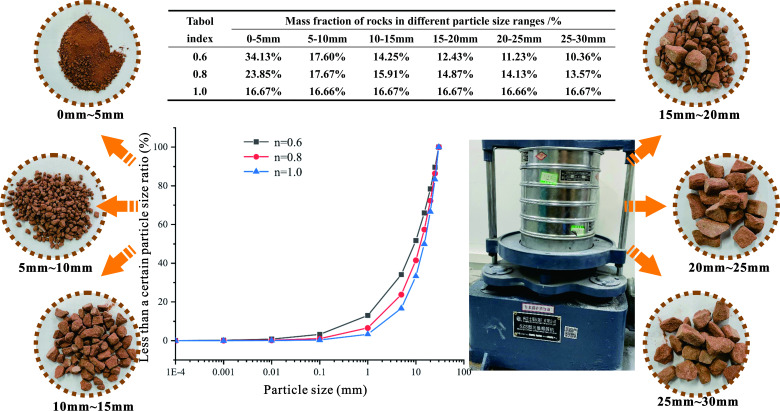
Screening of fractured rock masses with different gradations.

In this experiment, broken sandstone was selected as the filling material. According to Li Xingshang’s results on the particle size distribution of fractured rock masses (medium hard rocks) in the caving zone, the theory of fractured rock classification was used to fit it [36]. The fitting effect was better when broken gradations *n* = 0.6, 0.8, and 1.0 were obtained. Finally, the samples were weighed and grouped according to the grading required for the experiment (figure 3).

### 2.3. Experimental schemes and testing methods

#### 2.3.1. Physical and mechanical properties test of loess-based slurry material

To study the influence of the material ratio on the physical and mechanical properties of loess-based grout, three influencing factors, namely the water-to-solid ratio, solid-to-solid ratio and water glass content, were selected for the experiment. Five different values were taken for each factor, and parallel experiments were conducted on each group of samples. Fifteen single-factor experimental schemes were designed (table 3). Three parallel samples were made for each experiment, and the average of the results was taken. If there is significant dispersion, abnormal data will be removed and additional experiments will be conducted to ensure data reliability.

**Figure 4.**
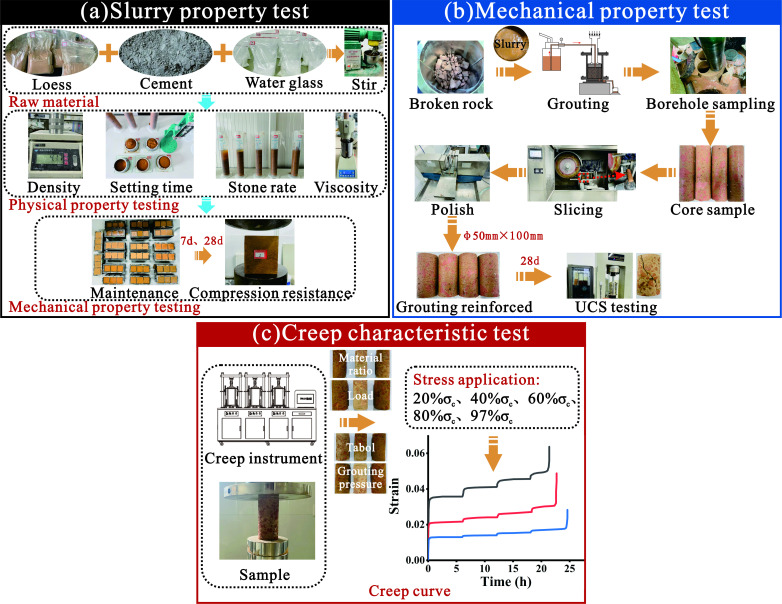
Experimental design.

Before the experiment, the mass of each component was calculated and weighed based on the moisture content of the loess sample determined by the drying method and the slurry ratio listed in table 3. When preparing the slurry, water was poured into the loess and mixed thoroughly using an NJ−160B cement slurry mixer. The cement was then poured into batches and mixed to ensure that the cement particles were fully wrapped around the loess. Finally, a water glass additive was added to promote the hydration reaction of the grouting material. After continuous stirring for 3−5 minutes until the slurry became colloidal, various properties of the loess-based slurry material were measured (figure 4a).

**Table 3.** Design of testing scheme for slurry characteristics.

test number	water-solid ratio	cement (loess) content /%	water glass content /%
1	1:0.6	30 (70)	3
2	1:0.8	30 (70)	3
3	1:1.0	30 (70)	3
4	1:1.2	30 (70)	3
5	1:1.4	30 (70)	3
6	1:1.0	10 (90)	3
7	1:1.0	20 (80)	3
8	1:1.0	30 (70)	3
9	1:1.0	40 (60)	3
10	1:1.0	50 (50)	3
11	1:1.0	30 (70)	1
12	1:1.0	30 (70)	2
13	1:1.0	30 (70)	3
14	1:1.0	30 (70)	4
15	1:1.0	30 (70)	5

#### 2.3.2. Grouting reinforcement strength characteristic test

To study the reinforcement effect of loess grout on fractured sandstone and the mechanical properties of the grouting reinforcement material, four influencing factors, namely loess grout ratio (factor A), Tabol gradation (factor B), overlying load (factor C) and grouting pressure (factor D), were selected for the experiment. Three different levels were used for each factor in the experiment, and 12 experimental schemes were designed (table 4). Three parallel samples were made for each experiment, and the average of the results was taken. If there is significant dispersion, abnormal data will be removed and additional experiments will be conducted to ensure data reliability.

**Table 4.** Design of testscheme for strength characteristics of grouting and solids. Note: (A) table 5. (B) *n* = 0.6, 0.8, 1.0. (C) 1.27, 2.55, 3.82 MPa. (D) 0.1, 0.3, 0.5 MPa.

number	loess slurry ratio (A)	tabol index (B)	overburden load (C)	grouting pressure (D)
1	A1	B2	C2	D2
2	A2	B2	C2	D2
3	A3	B2	C2	D2
4	A2	B1	C2	D2
5	A2	B2	C2	D2
6	A2	B3	C2	D2
7	A2	B2	C1	D2
8	A2	B2	C2	D2
9	A2	B2	C3	D2
10	A2	B2	C2	D1
11	A2	B2	C2	D2
12	A2	B2	C2	D3

**Table 5.** Proportioning ratio of loess grouting materials.

number	water-solid ratio	solid ratio	water glass /%	28d compressive strength /MPa
R1	1:1.0	3:7	3	2.10
R2	1:1.0	4:6	3	2.11
R3	1:1.2	3:7	3	2.36

Because the underground goaf is a closed space surrounded by unmined coal (rock) layers, from a macroscopic perspective, the stress process of the fractured rock mass in the goaf collapse zone during the compaction process of the overlying load is equivalent to the lateral compression process [37]. Therefore, this experiment simulates the grouting process under lateral compression conditions.

Before the grouting test began, the fractured rock mass was evenly placed into the cylinder assembly coated with Vaseline according to the calculated grading (*n* = 0.6, 0.8, 1.0), covered with the pressure-head assembly, and then the pressurized grouting test device was placed on the servo press. The slurry used in the experiment was prepared according to the ratio determined by the loess-based slurry material characteristic test described in §2.3.1. The prepared slurry was poured into the grouting bucket for mixing, connected to the grouting channel, the slurry inlet valve was opened, and injected into the crushed rock mass pressure grouting test device through the grouting pump. The selection of the test parameters and the basis for the selection are listed in table 6. After grouting was completed and cured for 72 hours, the grouting bucket model device was opened, and the grouting reinforcement material inside the model was removed, cut and polished. Finally, a solid sample with ϕ = 50 mm and h = 100 mm is obtained (figure 4*b*).

**Table 6.** Selection of parameters for grouting test [36,38,39].

parameters	selection criteria	parameter selection	notes
overburden load	the vertical geostress at depths of 50 m, 100 m and 150 m were selected for the experiment (with a rock density of 2600 N/m^3^)	1.27 MPa 2.55 MPa 3.82 MPa	the axial load of fractured rock mass mainly comes from the self-weight stress of the overlying rock mass
grading	according to Li Xingshang’s results on the particle size distribution of fractured rock masses (medium hard rocks) in the caving zone, the theory of fractured rock classification is used to fit it	0.6 0.8 1.0	the collapse of the roof and the fragmentation of the rock mass vary in particle size and shape
slurry ratio	injectability: Plastic viscosity range of 15–27 mPas Liquidity: Initial setting time greater than 2 hours, final setting time between 12 hours and 48 hours Consolidation performance: The stone formation rate is greater than 85%, the strength of the slurry material is greater than 2 MPa	Refer to §3.1	the grouting material needs to have injectability, certain compressive strength, etc
grouting pressure	permeation grouting is a method of filling with relatively small grouting pressure without significantly altering the original structure and volume of the fractured rock mass	0.1 MPa 0.3 MPa 0.5 MPa	if the grouting pressure is too high, there may be slurry leakage or seepage, while if the grouting pressure is too low, the diffusion range will be small

#### 2.3.3. Creep characteristic test of grouting reinforcement body

The grouting reinforcement material was placed on the KYSR-S servo-loaded creep test bench, and an axial load was applied to the specimen. According to the average uniaxial compressive strength of 20, 40, 60, 80 and 97% of the grouting reinforcement material of loess, the load values for graded loading in creep tests are taken, with a loading rate of 0.02 kN/s for each level. When the axial creep deformation rate was less than 0.001 mm/h, each load level was gradually increased until the specimen failed (figure 4c). Three parallel samples were made for each experiment, and the average of the results was taken. If there is significant dispersion, abnormal data will be removed and additional experiments will be conducted to ensure data reliability.

## 3. Results

### 3.1. Physical and mechanical properties of loess-based grout material

**Figure 5.**
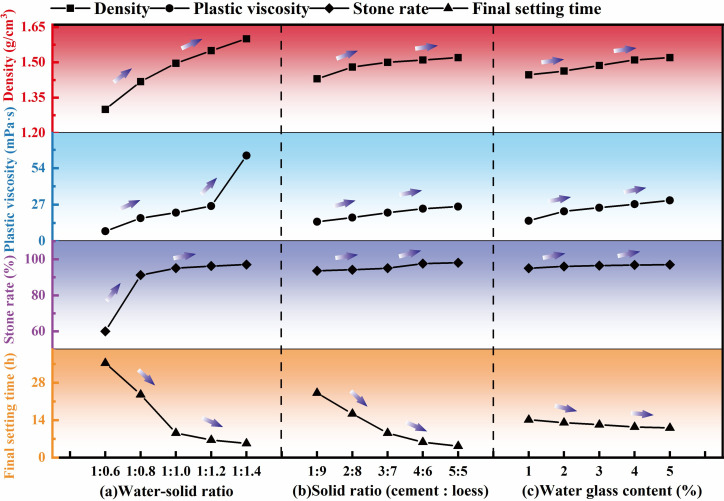
Performance test results of grouting materials. Note: (a) Solid ratio: 3:7; water glass: 3%. (b) Water–solid ratio of 1:1, water glass 3%. (c) Water-to-solid ratio of 1:1 and solid ratio of 3:7.

**Figure 6.**
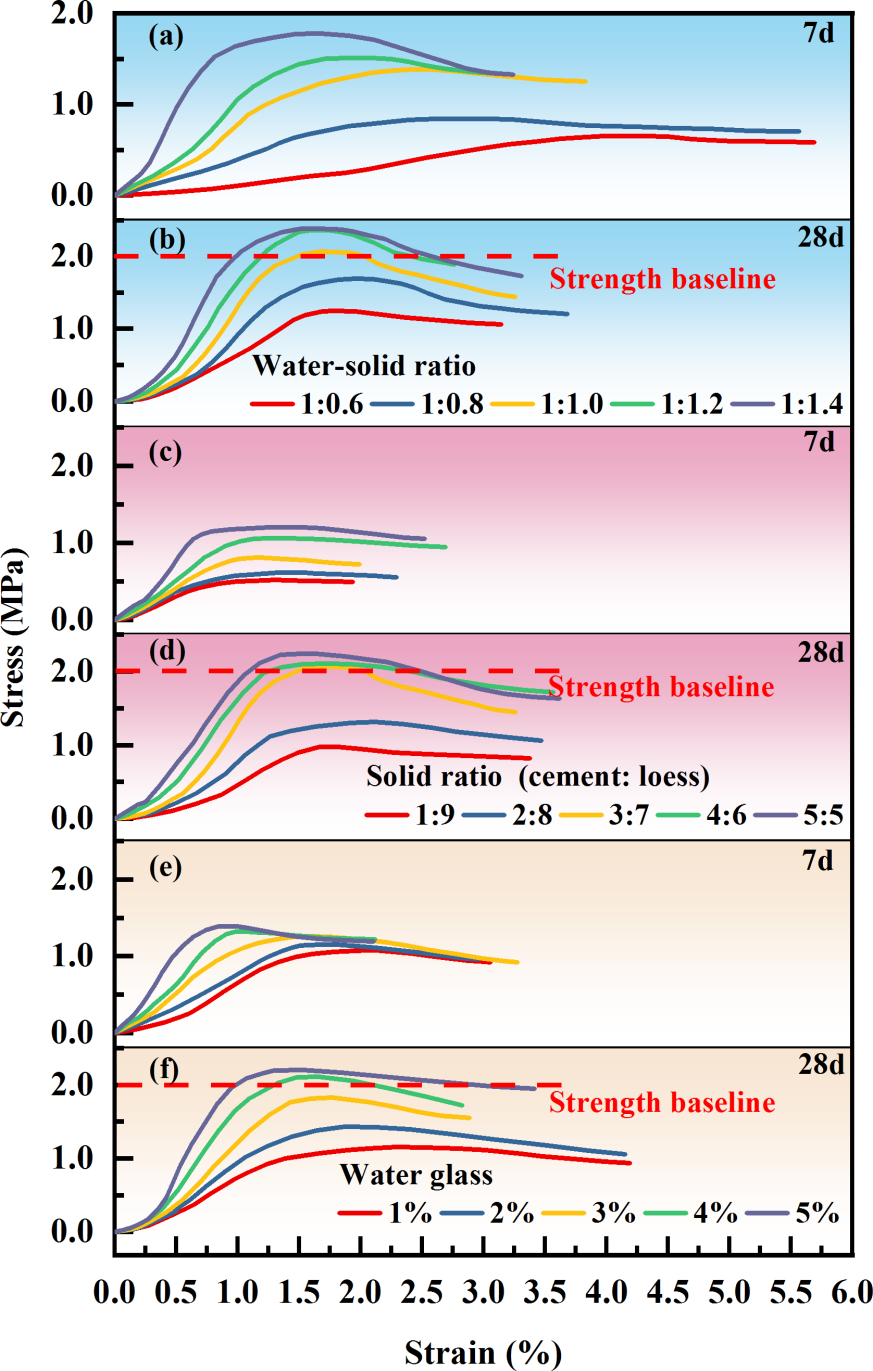
Stress–strain curve of grouted stone body. Note: (a)–(b) Solid ratio 3:7, water glass 3%. (c)–(d) Water-solid ratio 1:1, water glass 3%. (e)–(f) Water-solid ratio 1:1, solid ratio 3:7.

As the water-to-solid ratio decreased, the density, plastic viscosity, stone formation rate and early and late compressive strength of the grouting solid body increased, whereas the water separation rate and setting time gradually decreased (Figures 5a and 6a,b). The uniaxial compression curve of the grouting solid body includes a stage of ‘void compaction-elastic deformation-unstable development-failure’. As the water-to-solid ratio increased, the void compaction stage became more pronounced, and the elastic modulus decreased, resulting in a lower ultimate failure strength. This is because when the water-to-solid ratio is high, the free water content is high, the slurry is diluted, the hydration reaction cannot proceed fully, the viscosity of the slurry decreases, the settling rate of the solid particles increases, the water separation rate of the slurry increases and the stone formation rate decreases. The arrangement density of the solid substances decreases, leading to a decrease in the strength of the grouting solid body.

As the solid ratio increased, the density, viscosity, stone formation rate and early- and late-stage uniaxial compression strength of the grouting solid body increased, while the water separation rate and setting time decreased accordingly (figures 5b and 6c,d). This is because, as the solid phase ratio increased, the content of smaller cement particles in the slurry increased, and the solid phase substances fully contacted each other, promoting the hydration reaction of the slurry material and increasing the viscosity of the slurry. On the other hand, some particles in loess and cement undergo ionization during hydrolysis, resulting in cement and clay particles carrying positive and negative charges, respectively. This kind of charge force causes the loess and cement particles to generate charge exchange, thus accelerating the formation of gel from the slurry agglomeration, increasing the speed of gel formation and reducing the setting time. The interior of the grouting solid body is dense and has good integrity, which ultimately increases the compressive strength of the grouting solid body.

With the increase in water glass content, the density, plastic viscosity, stone formation rate and early and late uniaxial compressive strength of the grouting solid body increased, whereas the water separation rate and setting time gradually decreased (figures 5c and 6e,f). This is because sodium silicate reacts with the grouting material to produce silicate gel solidified products and silica compounds, which accelerates the curing reaction of the slurry, promotes water precipitation of the slurry, improves the stone rate, plastic viscosity and deformation resistance of the slurry and shortens the setting time of the slurry.

With a water-solid ratio of 1:1 and a solid ratio of 2:8, the 7 days compressive strength of traditional cement-fly ash grouting material is 0.33 MPa, and the 28 days compressive strength is 0.97 MPa [40]. Under the same proportioning conditions, the compressive strength of the loess-based grouting material studied in this article reached 0.66 MPa at 7 days and 1.25 MPa at 28 days, indicating that its mechanical properties are significantly better than traditional cement-based grouting materials, with stronger compressive and deformation resistance.

According to the results of the loess grouting material ratio test, the ratio that satisfied the filling performance of the grouting material was selected in the single-factor test (table 5).

### 3.2. Mechanical properties of grouting reinforcement material in loess base

**Figure 7.**
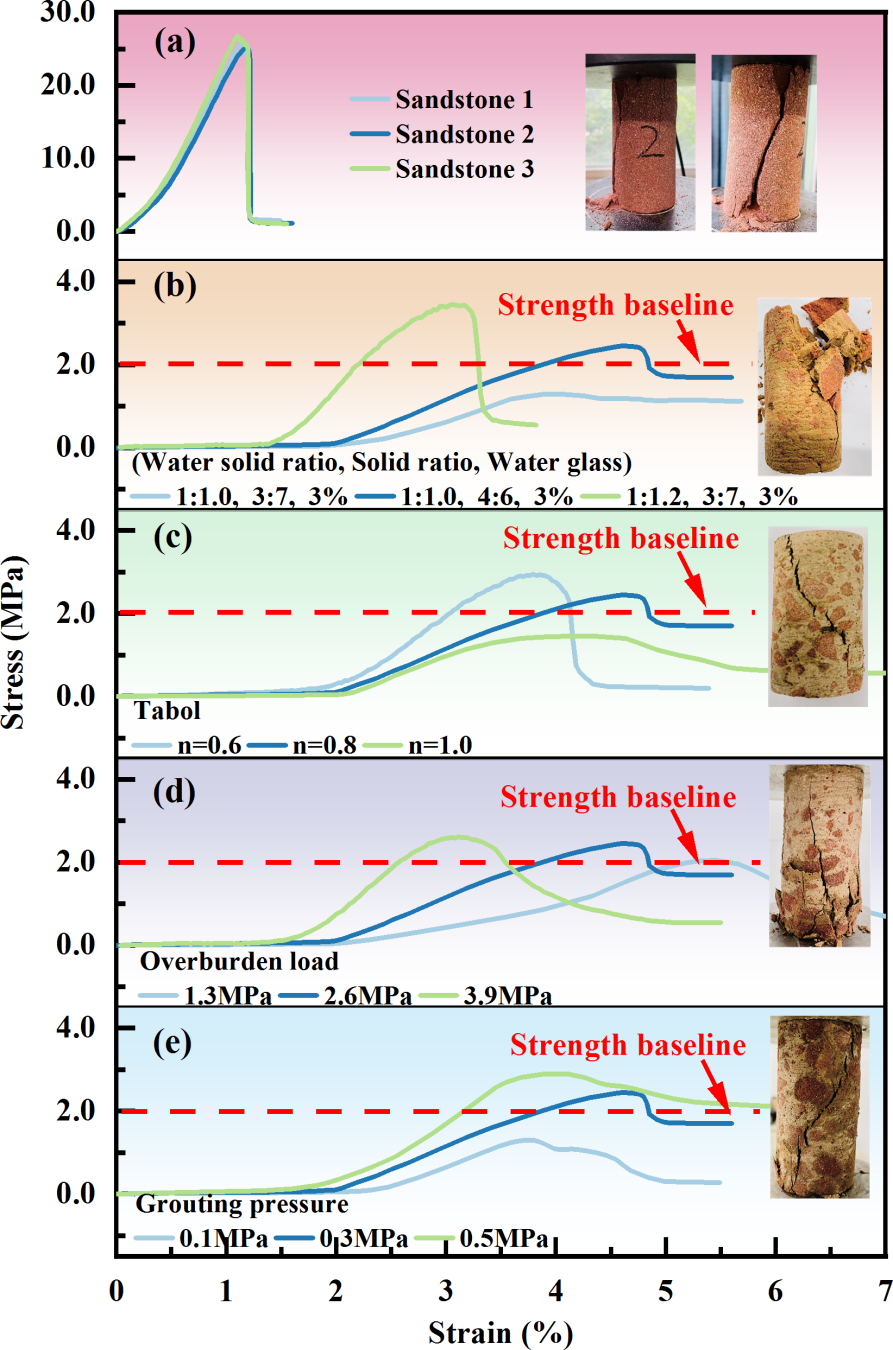
Stress–strain curve of grouting reinforcement material. Note: (b) *n* = 0.8; overburden load = 2.6 MPa, grouting pressure = 0.3 MPa. (c) R2, overburden load of 2.6 MPa, grouting pressure of 0.3 MPa. (d) R2, *n* = 0.8, grouting pressure = 0.3 MPa. (e) R2, *n* = 0.8; overburden load = 2.6 MPa.

The stress–strain curves of the intact sandstone and grouting reinforcement material under uniaxial compression are shown in figure 7. It can be concluded from the figure that the intact sandstone and grouting reinforcement material specimens under different influencing factors undergo stages of void compaction, elastic deformation, yield, failure and residual deformation after the peak during uniaxial compression.

(1) Void compaction stage: As shown in figure 7a, the intact sandstone samples had some natural micropores, but their overall density was high, and the internal fracture development was relatively low, resulting in an indistinct void compaction stage for the original rock. By contrast, as shown in figure 7b,e, the void compaction stage of the grouting reinforcement samples was more pronounced than that of the original rock. This stage represents the ‘breaking-in phase’ between the fractured rock mass and the slurry material, where the early stress–strain curves of the samples exhibit a ‘broken line’ characteristic. This is because the interior of the grouting reinforcement samples was not completely filled with slurry, leaving many voids compared to the intact rock samples. During the initial loading process, the portions not filled with slurry gradually closed, causing the stress–strain curve of the samples to display a concave shape. Therefore, in the initial compaction stage, the axial strain of the grouting reinforcement samples was relatively large and more pronounced than that of the original rock. Additionally, owing to the influence of the slurry mix ratio, crushed stone gradation, overlying load and grouting pressure, there are certain differences among the reinforcement materials. As the strength of the slurry decreased, the filling effect of the grouting became poorer, and the bonding force weakened, making the void compaction stage more distinct.
(2) Elastic deformation stage: After the initial compaction stage, the intact sandstone samples became more homogeneous and dense, primarily exhibiting elastic deformation during compression, with the strain showing a linear increase as the load was applied. However, the grouting reinforcement material after compaction is the joint bearing force of the rock block and slurry material at this stage, and its compressive characteristics are the result of the coupling effect between the two. Compared with the original rock, it is less homogeneous and enters the yield stage after experiencing a relatively short elastic stage. As the strength of the grout increases, the grouting filling effect improves, the bonding strength is enhanced, the elastic stage of the grouting reinforcement material becomes more obvious and the linear slope increases.
(3) Yield stage: Owing to the good homogeneity of the intact sandstone, the microcracks within the rock samples developed stably, and the slope of the yield stage curve was similar to that of the elastic stage, still exhibiting a linear growth trend. In contrast, the reinforcement samples consisted of fractured rock mass and slurry material. When the internal stress concentration reaches a certain level, the coupling effect between the two weakens, resulting in the formation of cracks within the rock mass, slurry and bonding interface between the two. This leads to a significant change in the slope of the curve, transitioning from linear to nonlinear.

(1) Failure stage: After the intact sandstone samples reached the yield limit, internal cracks developed, expanded and connected, and the degree of fracture continuously increased. Compared with the original rock, the overall density of the grouting reinforcement material is poor; therefore, the peak strength at failure is low, and the overall compression deformation is large.
(2) Postpeak residual deformation stage: After the cracks in the intact sandstone samples developed and penetrated, the strength of the sample rapidly decreased from the peak value to a lower stress level, with the failure mode primarily exhibiting brittle failure. In contrast, owing to the evident plastic characteristics of the loess-based grouting reinforcement body, the grouting reinforcement samples primarily exhibited ductile failure. After failure, the samples retained some residual strength compared with the original rock, and as the axial strain increased, the stress remained within a relatively stable range.

**Figure 8.**
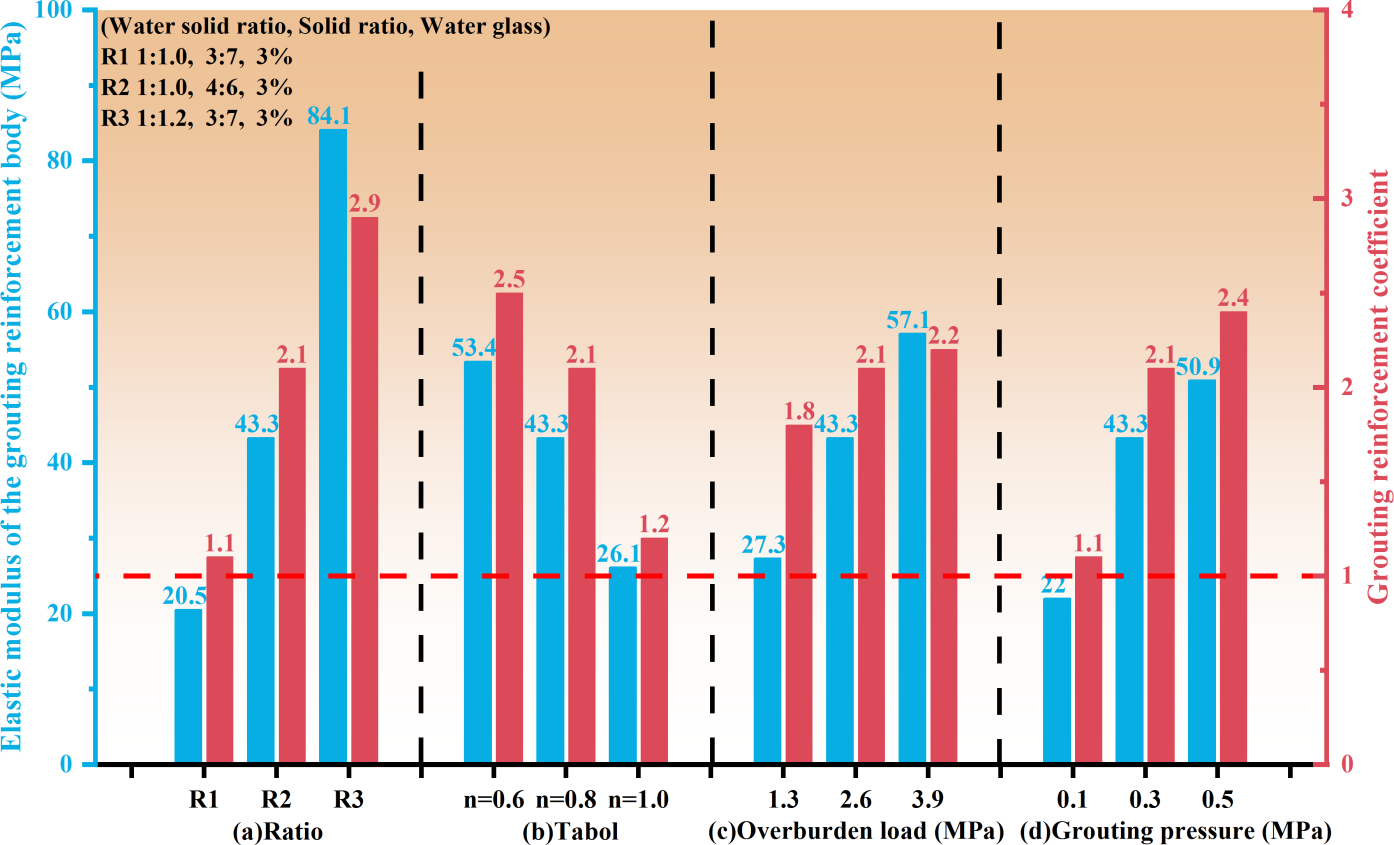
Strength characteristics of grouting reinforcement material. Note: (a) *n* = 0.8; overburden load = 2.6 MPa, grouting pressure = 0.3 MPa. (b) R2, overburden load = 2.6 MPa, grouting pressure = 0.3 MPa. (c) R2, *n* = 0.8, and grouting pressure = 0.3 MPa. (d) R2, *n* = 0.8, overburden load = 2.6 MPa.

The reinforcement coefficient is the ratio of the uniaxial compressive strength of the grouting reinforcement body to the postpeak residual strength of the original rock. Based on the test results shown in figure 7, it can be concluded that the grouting reinforcement coefficient was closely related to the slurry mix ratio, rock mass gradation, overlying load and grouting pressure (figure 8). As the slurry material ratio increased, the reinforcement coefficient gradually increased, indicating that the strength and viscosity of the loess slurry material increased, which improved the reinforcement effect of the fractured rock mass. As the grading of the fractured rock mass increased, the proportion of large crushed stones increased. Although large-sized crushed stones support the stacking skeleton, the surface area of the bonding contact between them and the slurry material is relatively large, and the compactness is poor. Therefore, the strength of the grouting reinforcement material decreased, and the reinforcement effect was relatively poor. The larger the overlying load, the stronger the coupling and bonding effect between the slurry and rock block. The water solvent in the slurry can quickly precipitate, increasing the stone formation rate and density of the grouting reinforcement material, thus enhancing the reinforcement effect. With an increase in grouting pressure, the slurry is more prone to diffusion, and the amount of slurry entering the voids increases. The filling density of the fractured rock mass increased, and the reinforcement effect was enhanced.

**Figure 9.**
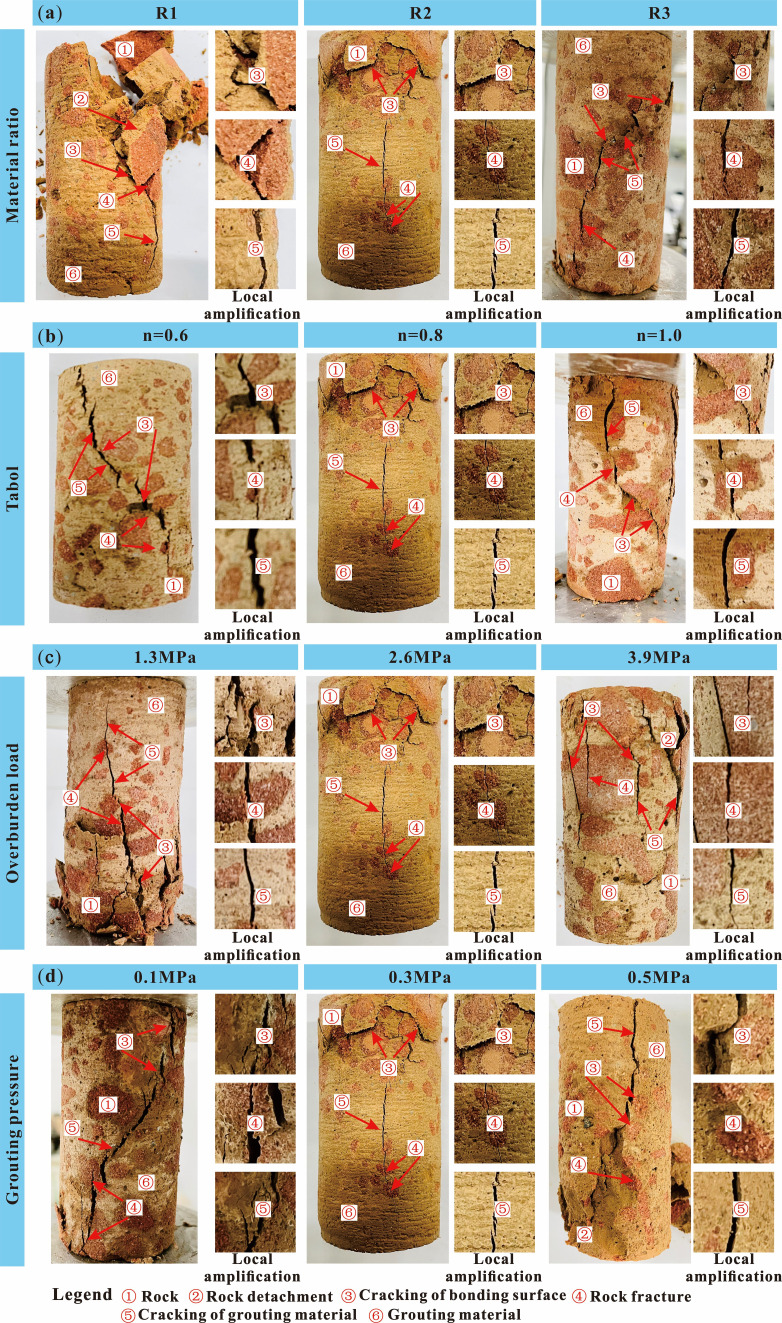
Failure modes of grouting reinforcement material. Note: (a) n=0.8; overburden load = 2.6 MPa, grouting pressure = 0.3 MPa. (b) R2, overburden load = 2.6 MPa, grouting pressure = 0.3 MPa. (c) R2, n = 0.8, and grouting pressure = 0.3 MPa. (d) R2, n = 0.8, overburden load = 2.6 MPa.

Compared with the intact sandstone samples, the loess-based grouting reinforcement samples exhibited several similarities in the failure modes during compression. Their cracks developed frequently and were relatively fine, making the failure mode more complex (figure 9). This phenomenon became more pronounced as the slurry strength decreased, rock mass gradation increased, overlying load decreased and grouting pressure decreased.

From the failure mode of the grouting reinforcement samples shown in figure 9, it can be seen that most of the internal cracks in the grouting reinforcement samples occurred at the junction between the rock blocks and the slurry material. This is because the compression process of the grouting reinforcement material is actually the process of the rock block and grout bearing the force together, and the compressive characteristics it shows are the result of the coupling of the two. However, owing to the influence of their respective mechanical properties, rock blocks and grout materials do not deform synchronously; therefore, it is easy to experience dislocation and delamination at the point where they are bonded, leading to stress concentration and the formation of cracks in the solid.

From the locally enlarged section in figure 9, it can be observed that the cracks in the reinforcement body primarily extend along the ‘rock-slurry-rock’ bonding interface, with some extension occurring at the slurry stone body, while the extension of cracks through the rock blocks is relatively rare. This is because the extension and expansion of cracks exhibit a certain ‘weakening’ characteristic. The strength of the bonding surface and the strength of the grouted stone body between the grout material and rock block, which have significant differences in mechanical deformation characteristics, are weaker than the strength of the rock block and are more suitable for the development of cracks. The internal homogeneity of the rock block was relatively good, resulting in fewer fractures passing through it.

Under different influencing factors, the greater the difference in mechanical deformation characteristics between the grout and rock blocks, the more likely it is to induce the formation of cracks, and the more blocks will fall off during failure. When the grading is small, the small particle size broken rock mass accounts for a high proportion and accumulates more closely. With the increase of grouting pressure, the grout can fully fill the pores of the broken rock mass, forming a good cementation structure, which improves the overall strength of grouting reinforcement material. Therefore, the compression failure mode of grouting reinforcement material sample shows a ‘rock-like’ characteristic, that is, the failure mode is mainly brittle splitting failure.

### 3.3. Creep characteristics of grouting reinforcement material in loess base

**Figure 10.**
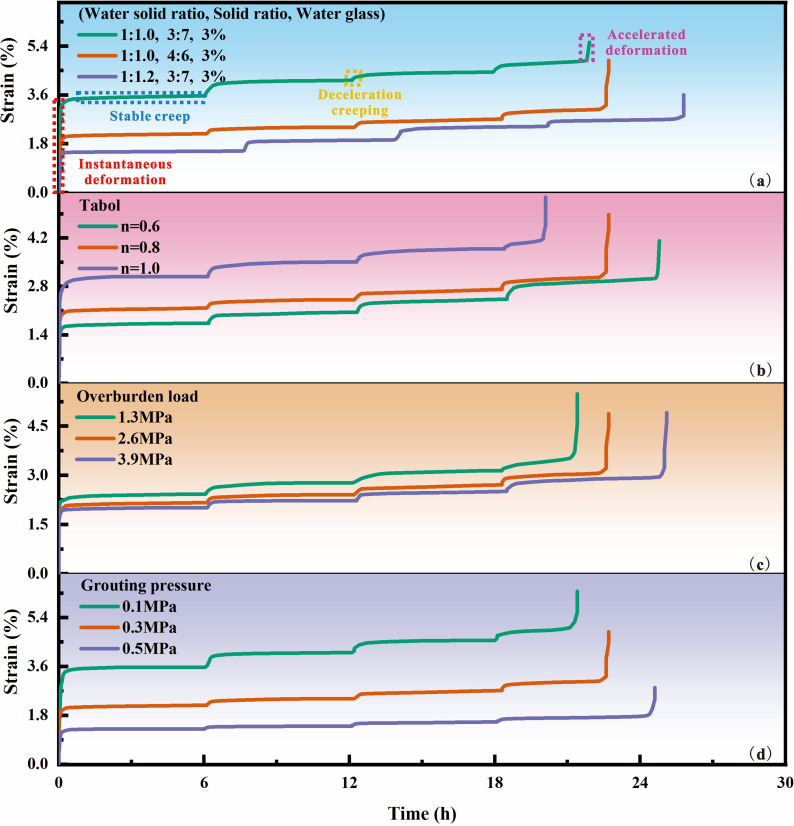
Creep characteristic curve of grouting reinforcement material. Note: (a) n = 0.8; overburden load = 2.6 MPa, grouting pressure = 0.3 MPa. (b) R2, overburden load = 2.6 MPa, grouting pressure = 0.3 MPa. (c) R2, n = 0.8, and grouting pressure = 0.3 MPa. (d) R2, n = 0.8, overburden load = 2.6 MPa.

Figure 10 shows the creep curves under graded loading of the reinforcement samples under different grout ratios, crushing gradations, overlying loads and grouting pressures. The creep test results in figure 10 show that: (i) with the application of axial load, the axial strain change trend of loess grouting reinforcement material samples is similar. (ii) The load and time of creep failure of the grouting reinforcement material increased gradually with an increase in the mortar ratio strength, decrease in broken gradation and increase in overlying load and grouting pressure. (iii) When the stress loading level was low, only the first two stages of creep occurred, namely deceleration creep and constant-velocity creep, and the deformation of the grouting reinforcement specimen tended to stabilize over time. As the stress level reached the failure strength, the grouting reinforcement specimen underwent an accelerated creep stage during loading, and the specimen failed owing to a sudden increase in creep rate.

Under the influence of the same factors, the instantaneous axial creep value of the grouting reinforcement samples was the highest after the application of the first load level (accounting for more than 60% of the final deformation). Subsequently, the instantaneous creep values after each loading level were lower than those at the first level. From the application of the second level of load onwards, as the load increased, the instantaneous creep values of the samples showed a growing trend at each load level. Under the same load level, the instantaneous creep value of the samples increased as the slurry strength decreased, gradation of the fractured rock increased, overlying load decreased and grouting pressure decreased. This is because the deformation of the grouting reinforcement under initial load mainly comes from the rapid closure of internal pores [41], resulting in a large instantaneous creep value. As the load continues to be applied, deformation is mainly caused by solid failure. Therefore, with the increase of load, the instantaneous creep value increases but is smaller than the initial instantaneous creep value. From a microstructural perspective, the slurry will form ettringite particles of different sizes and shapes on the surface of the fractured rock mass. As the grouting pressure increases, the contact between the slurry and the surface of the fractured rock mass becomes more complete, and the formation of sheet-like or clustered hydration products increases [41], effectively filling the pores and significantly reducing the porosity of the solid. At the same time, when the grading of the fractured rock mass is small, the proportion of small-sized crushed stones increases, and the accumulation becomes denser [42]. The bonding strength between the slurry and the fractured rock mass is enhanced, and the interface coupling effect and deformation coordination are improved, thereby significantly enhancing the overall creep resistance of the grouting reinforcement.

**Figure 11.**
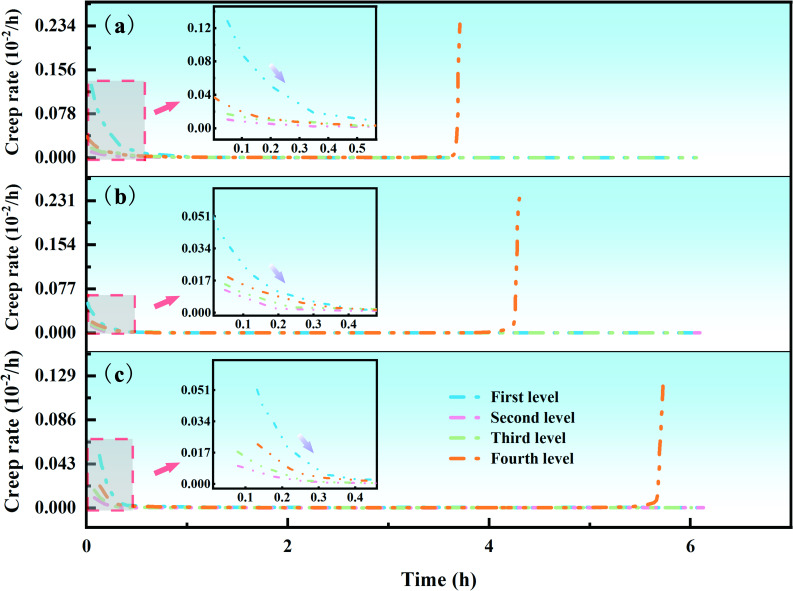
Creep rate curves of grouting reinforcement material with different material proportions. Note: n = 0.8,overburden load = 2.6 MPa, grouting pressure = 0.3 MPa.

To further investigate the uniaxial compressive creep performance of the loess-based grouting reinforcement bodies, the creep curve slopes of the reinforcement bodies at different moments under graded loading conditions were analysed. This analysis revealed the variation laws of creep rates at different stress levels and times. Consider the factors of different grouting material ratios as an example, as shown in figure 11. At each stress level, the creep rate trends of the loess-based grouting reinforcement bodies were similar. At the beginning of each load application, the creep rate of the samples was relatively high. As the creep time increased, the creep rate decreased rapidly and then stabilized, indicating a transition from a decelerating creep to a constant-rate creep stage. From the locally enlarged view, it can be observed that as the axial load level increased, the creep rate of the samples showed an increasing trend. When the axial load reached the long-term strength of the reinforcement body, the samples entered the accelerating creep stage after experiencing a period of constant-rate creep, at which point the creep rate began to increase sharply, leading to the failure of the reinforcement samples.

**Figure 12.**
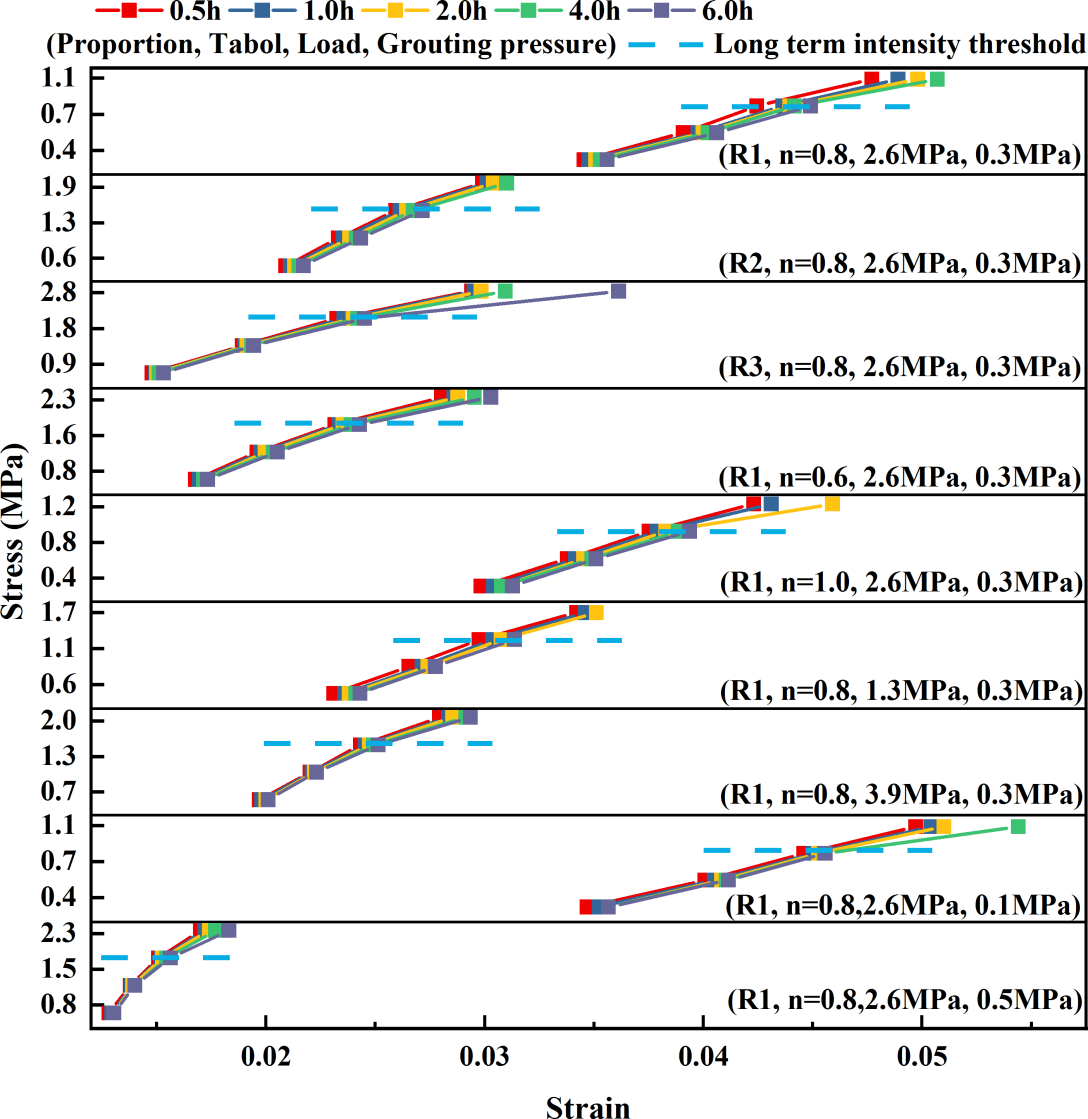
Stress–strain curve of grouting reinforcement material at constant time.

During the long-term creep process, the reinforcement body continued to experience damage and failure. The strength at which creep failure occurs is referred to as the long-term strength. There is a difference between this value and the instantaneous peak strength during direct compressive failure. Thus, it is not appropriate to evaluate the ability of a reinforcement body to resist creep failure using its peak strength. Currently, the methods for determining the long-term strength of the reinforcement body include direct, isochronous curve and transitional creep methods [43]. Among them, the isochronous curve method is the most common method for determining the long-term strength of a specimen, which is the relationship curve between creep deformation and stress at the same time point in multi-stage creep tests. When the stress level is lower than the long-term strength, the specimen only undergoes viscoelastic deformation, and creep deformation and stress show a nearly linear relationship. When the stress level is higher than the long-term strength, the internal damage and rupture of the specimen lead to the occurrence of viscoplastic deformation, and creep deformation shows a nonlinear relationship with stress [44]. Therefore, defining the inflection point of the linear to nonlinear transition of the isochronous curve cluster as the long-term intensity, this method can obtain more accurate long-term intensity values. Figure 12 shows the isochronous stress–strain curves of the loess-based grouting reinforcement samples, based on which a further comparative analysis of the long-term strength of loess-based grouting reinforcement bodies under different influencing factors was conducted.

From figure 12, it can be observed that the isochronous stress-strain curves of the loess-based grouting reinforcement samples exhibit similar characteristics under different influencing factors. Specifically, as the stress level increased, the isochronous stress-strain curves gradually transitioned from linear to nonlinear, showing distinct inflection points. For loess-based grouting reinforcement bodies under the influence of different slurry mix ratios, crushed stone gradations, overlying loads and grouting pressures, when the stress levels are below 0.8 MPa to 2.1 MPa, 0.9 MPa to 1.8 MPa, 1.3 MPa to 1.6 MPa and 0.8 MPa to 1.8 MPa, respectively, the isochronous stress-strain curves are nearly a cluster of straight line segments, indicating that the samples exhibit viscoelastic characteristics. However, when the stress levels exceed these thresholds, the isochronous stress-strain curves undergo significant changes, with the samples transitioning from viscoelastic to viscoelastic-plastic behaviour. To sum up, within the scope considered in this test, when the ratio of grouting materials for loess-based is 1:1.2, 3:7, 3% water glass, the grading of broken rock mass is 0.8, the overlying load is 2.6 MPa and the grouting pressure is 0.3 MPa, the long-term strength of grouting reinforcement material formed is the largest, reaching 2.1 MPa, accounting for 65–80% of its instantaneous strength.

## 4. Discussion

**Figure 13.**
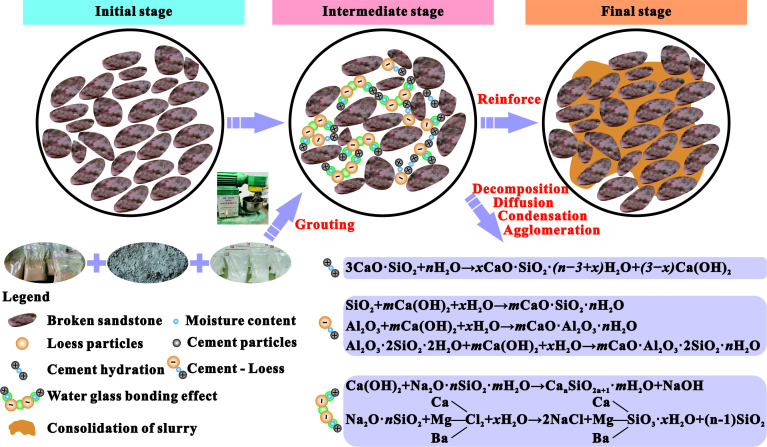
Analysis of cement-loess solidification reaction.

Studying the interparticle contact relationships and degree of hydration reaction in loess-based grouting reinforcement bodies aids in analysing the curing reaction mechanisms of the loess-cement slurry. The reinforcement of a loess-based slurry primarily relies on the hydration of cement. Through a series of physical and chemical processes, including decomposition, diffusion and dissolution, cement particles form a gel structure on their surfaces, leading to a denser stone body structure and thereby improving strength (figure 13). In contrast, larger loess particles acted as nucleation sites during the formation of the colloid. Some clay particles in the soil undergo hydrolysis and ionization, resulting in negatively charged clay particles that engage in a weak charge exchange with positively charged cement particles. This process causes clay particles to agglomerate and form a gel (figure 13).

After the addition of water glass to the loess-based slurry, most of the water glass reacts with the cement to form silica gel solidification products, whereas a small portion of the water glass reacts with the soil particles, creating silicate bridges between the soil particles (figure 13). Following a series of curing reactions, this process promotes the formation of a solid structure of the slurry and reduces the setting time. As the amount of cement increases, the intensity of the hydration process increases, the rate of gel formation accelerates, the setting time decreases and the distance between the particles shortens, leading to an increase in the structural strength.

**Figure 14.**
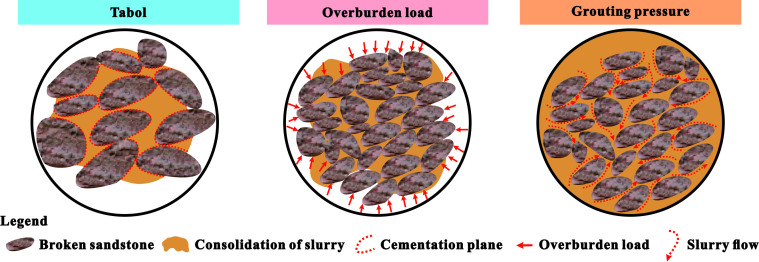
The reinforcement mechanism of grouting-reinforced loess under different influencing factors.

Grouting can effectively bond fractured rock masses into a whole. However, the compressive strength of the grouted solid varies under different influencing factors owing to the coupling effect between the grout and rock blocks (figure 14). As the water-to-solid ratio decreases, the solid ratio increases, the gradation decreases, the overlying load increases and the grouting pressure increases in the grout material ratio, the smaller the difference of mechanical deformation characteristics between the slurry material and the rock block, the better the overall strength and stability of grouting reinforcement material (figure 14), the failure mode of the final sample shows the ‘rock-like’ characteristics, the peak compressive strength and creep failure load of grouting reinforcement material increase and the axial creep variable gradually decreases. This is owing to the decrease in the water-to-solid ratio and the increase in the solid ratio in the mix, which leads to an increase in the bonding strength and strength of the slurry material. The grading of the fractured rock mass decreases with a higher proportion of small-sized crushed stones. The stacking between crushed stones and between crushed stones and grout is more compact, resulting in a higher strength after bonding. As the grading increases, the proportion of large crushed stones increases. Although large-sized crushed stones support the stacking skeleton, the surface area of the bonding contact between the large-sized crushed stones and grout material is relatively large. The compressive strength of the grouted solid mainly depends on the bonding strength between the grout material and rock block. Therefore, the strength of the grouting reinforcement material with larger grading is lower. As the overlying load increases, the contact between the grout and fractured rock mass during bonding becomes tighter, and the strength increases. The greater the grouting pressure, the higher the degree of pore filling inside the fractured rock mass, and the slurry is relatively full. The contact between the slurry material and the fractured rock mass is tighter, and its compressive strength increased.

It should be pointed out that the experimental results in this manuscript were obtained under indoor conditions of normal temperature, pressure and humidity. The parameters used, such as the ratio of loess-based grouting materials, the grading of fractured rock masses, the overlying load, and grouting pressure, were all set based on grouting conditions and have not yet covered the complex and variable underground environment in actual engineering. Therefore, the subsequent research will further consider the impact of environmental factors such as groundwater and temperature on the mechanical properties and creep behaviour of grouting reinforcement material, so as to improve the applicability and reliability of the research results in actual projects.

## 5. Conclusion

This study focused on the mechanics and creep characteristics of loess grouting reinforcement materials in a goaf. Through the slurry ratio test, grouting reinforcement material mechanics and creep test, the strengthening mechanism of grouting reinforcement material under different influence factors, the failure mode of ‘rock-slurry-rock’ structural plane and the creep deformation characteristics are studied. The main conclusions are as follows.

(1) As the water-to-solid ratio increases, the solid-to-solid ratio decreases and the amount of water glass decreases. The water separation rate and setting time of the loess-based slurry increase, while the density, plastic viscosity, stone formation rate and compressive strength of the grouting solid body decrease. When the water-to-solid ratio is controlled at 1:1.4–1:1.0, the solid ratio is controlled at 3:7−5:5, the water glass content is 3−5%, and the flowability, consolidation performance, setting time and compressive strength of the slurry meet the requirements for grouting treatment in the goaf.
(2) The compressive properties of the grouting reinforcement material are the result of the coupling effect between the rock block and grout. With the increase in grout strength, reduction in crushing gradation, increase in overlying load and increase in grouting pressure, the difference in mechanical deformation characteristics between the grout and rock block decreases, the strength of the grouting plus solid and the grouting reinforcement coefficient increase and the compression failure pattern shows the ‘rock-like’ characteristics. Owing to the characteristic of ‘weakening’ in the extension and expansion of cracks, during solid failure, cracks mainly propagate along the ‘rock-slurry-rock’ bonding surface, followed by extension and expansion along the grouting solid body, while the expansion of cracks that penetrate the rock block is relatively small.
(3) As the strength of the grout increases, the crushing gradation decreases, the overlying load increases, the grouting pressure increases and the creep failure load and failure time of the grouting reinforcement material gradually increase. Before the creep failure of the specimen, with the increase in axial loads at all levels, the instantaneous strain and axial creep values of the grouting reinforcement material first decrease and then increase. The creep rate of the grouting reinforcement material shows a trend of rapid decrease followed by stabilization. In the accelerated creep stage, the creep rate increases sharply. The long-term strength of the loess grouting reinforcement material is 2.1 MPa, which is 65−80% of the instantaneous strength of the sample.

## Data Availability

All used data are included in the electronic supplementary material [45].
